# Supplementary material for: Bioinformatics core competencies for undergraduate life sciences education
Source: PLoS One. 2018 Jun 5;13(6):e0196878. doi: 10.1371/journal.pone.0196878 (PMC5988330; doi:10.1371/journal.pone.0196878)
Supplement: S1 Survey — As explained in the narrative, the survey was branched, with some questions or sections presented or skipped depending on the responses to previous, filtering questions. In this view, the branching structure of the survey can be followed. (PDF) [file pone.0196878.s001.pdf]

Integrating Bioinformatics into Life Sciences Education

Default Question Block — Block Options

Q46

Dear Life Sciences Educator,

The Network for Integrating Bioinformatics into Life Sciences Education (NIBLSE; "nibbles") is a National Science Foundation Research Coordination Network for Undergraduate Biology Education (RCN-UBE) devoted to establishing bioinformatics as essential to the undergraduate life sciences curriculum. To that end, we are asking the community to help us determine core bioinformatics competencies for the undergraduate curriculum.

We are asking you to complete a short, anonymous survey if you are in one or more of the following groups:

- Educators who teach undergraduate life sciences at a 2-year or 4-year college, university, or technical school.
- Educators who supervise graduate students and who expect, or would like to expect, graduate student familiarity with bioinformatics.
- Biologists and/or bioinformaticians who teach/provide training in bioinformatics as part of their work at a company or organization, but not as part of a for-credit course at a college or university.

The survey should take you approximately 15 minutes to complete.

We invite you to read more about our activities and other ways to contribute and provide feedback at our project website or contact us at the address below. Thank you in advance for your input.

NIBLSE Leadership Team:
Mark Pauley (mark@niblse.org), University of Nebraska at Omaha
Elizabeth Dinsdale, San Diego State University
William Morgan, College of Wooster
Anne Rosenwald, Georgetown University
Eric Triplett, University of Florida

This survey is covered by IRB 161-16-EX. The survey administrator will disassociate any linked or uploaded files from your survey response before sharing these with the research team. For questions about the survey, please contact Mindy McWilliams. NIBLSE is supported by NSF Award #1539900.

NIBLSE is a proud partner of QUBES: https://qubeshub.org/

- ◯ I agree to participate.
- ◯ I do not agree to participate.

Condition: I do not agree to participate. Is Selected. Skip To: End of Survey.

Page Break

Q51

To begin the survey, please select the statement that best describes your view of bioinformatics in undergraduate life sciences education.

- I think bioinformatics should be integrated into undergraduate life sciences education.
- I do not think bioinformatics should be integrated into undergraduate life sciences education.

Condition: I do not think bioinformati... Is Selected. Skip To: ABOUT YOU.

Page Break

Q57

Please select the statement below that best describes you.

- I teach at a 4-year college or university.
- I teach at a 2-year college or technical school.
- I teach/provide training in bioinformatics as a regular part of my work at a company or organization, but not as part of a for-credit course at a college or university.

Condition: I teach/provide training in... Is Selected. Skip To: ABOUT YOUR TRAINING.

Q1

Please select the statement below that best describes your current teaching of bioinformatics content at your institution.

- I teach dedicated bioinformatics course(s) to undergraduates majoring in life sciences or closely related disciplines.
- I teach undergraduates majoring in life sciences or closely related disciplines and include substantial bioinformatics (more than one lecture/lab section) in my teaching.
- I teach undergraduates majoring in life sciences or closely related disciplines and DO NOT currently include substantial bioinformatics (more than one lecture/lab section) in my teaching but will/would like to do so in the future.
- I supervise graduate students in the life sciences or closely related disciplines and expect, or would like to expect, graduate student familiarity with bioinformatics.

Page Break

Display This Question:

If Please select the statement below that best describes your current teaching of bioinformatics con... I teach dedicated bioinformatics course(s) to undergraduates majoring in life sciences or closely related disciplines. Is Selected

Or Please select the statement below that best describes your current teaching of bioinformatics con... I teach undergraduates majoring in life sciences or closely related disciplines and include substantial bioinformatics (more than one lecture/lab section) in my teaching. Is Selected

Or Please select the statement below that best describes your current teaching of bioinformatics con... I teach undergraduates majoring in life sciences or closely related disciplines and DO NOT currently include substantial bioinformatics (more than one lecture/lab section) in my teaching but will/would like to do so in the future. Is Selected

Or Please select the statement below that best describes your current teaching of bioinformatics con... I supervise graduate students in the life sciences or closely related disciplines and expect, or would like to expect, graduate student familiarity with bioinformatics. Is Selected

Q42

ABOUT YOUR CURRICULUM

Display This Question:

If Please select the statement below that best describes your current teaching of bioinformatics con... I teach dedicated bioinformatics course(s) to undergraduates majoring in life sciences or closely related disciplines. Is Selected

And Please select the statement below that best describes you. I teach at a 4-year college or university. Is Selected

Q2

What is the level of the dedicated bioinformatics course(s) you teach? (Check all that apply.)

- Freshman
- Sophomore
- Junior
- Senior

Display This Question:

If Please select the statement below that best describes your current teaching of bioinformatics con... I teach undergraduates majoring in life sciences or closely related disciplines and include substantial bioinformatics (more than one lecture/lab section) in my teaching. Is Selected

And Please select the statement below that best describes you. I teach at a 4-year college or university. Is Selected

Q36

What is the level of the courses with bioinformatics content that you teach? (Check all that apply.)

- Freshman
- Sophomore
- Junior
- Senior

Display This Question:

If Please select the statement below that best describes your current teaching of bioinformatics con... I teach dedicated bioinformatics course(s) to undergraduates majoring in life sciences or closely related disciplines. Is Selected

And Please select the statement below that best describes you. I teach at a 2-year college or technical school. Is Selected

Or Please select the statement below that best describes your current teaching of bioinformatics con... I teach undergraduates majoring in life sciences or closely related disciplines and include substantial bioinformatics (more than one lecture/lab section) in my teaching. Is Selected

And Please select the statement below that best describes you. I teach at a 2-year college or technical school. Is Selected

Q59

What is the level of the course(s) you teach? (Check all that apply.)

- ☐ Freshman
- ☐ Sophomore

Display This Question:

If Please select the statement below that best describes you. I teach at a 2-year college or technical school. Is Selected

And Please select the statement below that best describes your current teaching of bioinformatics con... I teach undergraduates majoring in life sciences or closely related disciplines and DO NOT currently include substantial bioinformatics (more than one lecture/lab section) in my teaching but will/would like to do so in the future. Is Selected

Q60

What is the level of the courses you teach in which you would like to include bioinformatics content? (Check all that apply.)

- ☐ Freshman
- ☐ Sophomore

Display This Question:

If Please select the statement below that best describes your current teaching of bioinformatics con... I teach dedicated bioinformatics course(s) to undergraduates majoring in life sciences or closely related disciplines. Is Selected

Or Please select the statement below that best describes your current teaching of bioinformatics con... I teach undergraduates majoring in life sciences or closely related disciplines and include substantial bioinformatics (more than one lecture/lab section) in my teaching. Is Selected

Q56

As part of our work, we are building an online repository of bioinformatics syllabi and content assessments. Would you be willing to share your syllabus/syllabi and/or content assessment(s) with us so they could be added to this repository?

- ○ Yes
- ○ Maybe
- ○ No

Display This Question:

If Please select the statement below that best describes your current teaching of bioinformatics con... I teach undergraduates majoring in life sciences or closely related disciplines and DO NOT currently include substantial bioinformatics (more than one lecture/lab section) in my teaching but will/would like to do so in the future. Is Selected

Q37

What is the level of the courses you teach in which you would like to include bioinformatics content? (Check all that apply.)

- Freshman
- Sophomore
- Junior
- Senior

Display This Question:

If Please select the statement below that best describes your current teaching of bioinformatics con... I teach undergraduates majoring in life sciences or closely related disciplines and DO NOT currently include substantial bioinformatics (more than one lecture/lab section) in my teaching but will/would like to do so in the future. Is Selected

Q38

What is preventing you from including bioinformatics content in these courses?

Display This Question:

If Please select the statement below that best describes your current teaching of bioinformatics con... I teach undergraduates majoring in life sciences or closely related disciplines and DO NOT currently include substantial bioinformatics (more than one lecture/lab section) in my teaching but will/would like to do so in the future. Is Selected

Or Please select the statement below that best describes your current teaching of bioinformatics con... I supervise graduate students in the life sciences or closely related disciplines and expect, or would like to expect, graduate student familiarity with bioinformatics. Is Selected

Q40

Are there undergraduate courses with bioinformatics content at your institution that life sciences students routinely take? If "yes," please encourage those teaching these classes to complete the survey (forward the solicitation e-mail).

- Yes
- No
- Don't know

Display This Question:

If Please select the statement below that best describes your current teaching of bioinformatics con... I teach dedicated bioinformatics course(s) to undergraduates majoring in life sciences or closely related disciplines. Is Selected

Or Please select the statement below that best describes your current teaching of bioinformatics con... I teach undergraduates majoring in life sciences or closely related disciplines and include substantial bioinformatics (more than one lecture/lab section) in my teaching. Is Selected

Q4

Aside from the course(s) you teach, are there other undergraduate courses with bioinformatics content at your institution that life sciences students routinely take? If "yes," please encourage those teaching these courses to complete this survey (forward the solicitation e-mail).

- Yes
- No
- Don't know

Display This Question:

If Please select the statement below that best describes your current teaching of bioinformatics con... I teach dedicated bioinformatics course(s) to undergraduates majoring in life sciences or closely related disciplines. Is Selected

Or Please select the statement below that best describes your current teaching of bioinformatics con... I teach undergraduates majoring in life sciences or closely related disciplines and include substantial bioinformatics (more than one lecture/lab section) in my teaching. Is Selected

Or Please select the statement below that best describes your current teaching of bioinformatics con... I teach undergraduates majoring in life sciences or closely related disciplines and DO NOT currently include substantial bioinformatics (more than one lecture/lab section) in my teaching but will/would like to do so in the future. Is Selected

Q5

In your opinion, are additional undergraduate courses with bioinformatics content needed at your institution?

- Yes
- No
- Don't know

Display This Question:

If In your opinion, are additional undergraduate courses with bioinformatics content needed at your... Yes Is Selected

Q6

Optional: Please describe briefly; include any barriers to development and/or implementation.

Display This Question:

If Please select the statement below that best describes your current teaching of bioinformatics con... I teach dedicated bioinformatics course(s) to undergraduates majoring in life sciences or closely related disciplines. Is Selected

Or Please select the statement below that best describes your current teaching of bioinformatics con... I teach undergraduates majoring in life sciences or closely related disciplines and include substantial bioinformatics (more than one lecture/lab section) in my teaching. Is Selected

Or Please select the statement below that best describes your current teaching of bioinformatics con... I teach undergraduates majoring in life sciences or closely related disciplines and DO NOT currently include substantial bioinformatics (more than one lecture/lab section) in my teaching but will/would like to do so in the future. Is Selected

Or Please select the statement below that best describes your current teaching of bioinformatics con... I supervise graduate students in the life sciences or closely related disciplines and expect, or would like to expect, graduate student familiarity with bioinformatics. Is Selected

Q7 Is an undergraduate bioinformatics certificate or minor offered at your institution?

- Yes
- No
- Don't know

Display This Question:

If Is an undergraduate bioinformatics certificate or minor offered at your institution? Yes Is Selected

Q8 Optional: Please give certificate or minor name, department/unit in which it's offered, and website URL (if available).

Display This Question:

If Please select the statement below that best describes your current teaching of bioinformatics con... I teach dedicated bioinformatics course(s) to undergraduates majoring in life sciences or closely related disciplines. Is Selected

Or Please select the statement below that best describes your current teaching of bioinformatics con... I teach undergraduates majoring in life sciences or closely related disciplines and include substantial bioinformatics (more than one lecture/lab section) in my teaching. Is Selected

Or Please select the statement below that best describes your current teaching of bioinformatics con... I teach undergraduates majoring in life sciences or closely related disciplines and DO NOT currently include substantial bioinformatics (more than one lecture/lab section) in my teaching but will/would like to do so in the future. Is Selected

Or Please select the statement below that best describes your current teaching of bioinformatics con... I supervise graduate students in the life sciences or closely related disciplines and expect, or would like to expect, graduate student familiarity with bioinformatics. Is Selected

Q9 Is an undergraduate bioinformatics major offered at your institution?

- Yes
- No
- Don't know

Display This Question:

If Is an undergraduate bioinformatics major offered at your institution? Yes Is Selected

Q10

Optional: Please give name, department/unit in which it's offered, and website URL (if available):

Display This Question:

If Please select the statement below that best describes you. I teach/provide training in bioinformatics as a regular part of my work at a company or organization, but not as part of a for-credit course at a college or university. Is Selected

Q53

ABOUT YOUR TRAINING

Display This Question:

If Please select the statement below that best describes you. I teach/provide training in bioinformatics as a regular part of my work at a company or organization, but not as part of a for-credit course at a college or university. Is Selected

Q54

Briefly describe the format of the bioinformatics training (e.g., boot camp, short course, etc.) you most commonly provide.

Display This Question:

If Please select the statement below that best describes you. I teach/provide training in bioinformatics as a regular part of my work at a company or organization, but not as part of a for-credit course at a college or university. Is Selected

Q55

Briefly describe your audience for this training.

Display This Question:

If Please select the statement below that best describes you. I teach/provide training in bioinformatics as a regular part of my work at a company or organization, but not as part of a for-credit course at a college or university. Is Selected

Q43

In your opinion, what are the biggest bioinformatics needs of those taking your training?

Display This Question:

If Please select the statement below that best describes you. I teach/provide training in bioinformatics as a regular part of my work at a company or organization, but not as part of a for-credit course at a college or university. Is Selected

Q42

What reasons do your students provide as to why they are taking your training (e.g., professional advancement, have a research problem they need to solve, have been tasked with teaching a course in bioinformatics)?

Page Break

Q43

BIOINFORMATICS COMPETENCIES FOR UNDERGRADUATE LIFE SCIENTISTS

Q13

In your opinion, how important is it for undergraduates majoring in life sciences or closely related disciplines to...

	Not at all important	Slightly important	Moderately important	Very important	Extremely important	× No opinion
Understand the role of computation and data mining in hypothesis-driven processes within the life sciences?	○	○	○	○	○	○
Understand computational concepts used in bioinformatics, e.g., meaning of algorithm, bioinformatics file formats?	○	○	○	○	○	○
Know statistical concepts used in bioinformatics, e.g., E-value, z-scores, t-test?	○	○	○	○	○	○
Know how to access genomic data, e.g., in NCBI nucleotide databases?	○	○	○	○	○	○
Be able to use bioinformatics tools to						

	Not at all important	Slightly important	Moderately important	Very important	Extremely important	× No opinion
[illegible] analyze genomic data, e.g., BLASTN, genome browser?	○	○	○	○	○	○
Know how to access gene expression data, e.g., in UniGene, GEO, SRA?	○	○	○	○	○	○
Be able to use bioinformatics tools to analyze gene expression data, e.g., GeneSifter, David, ORF Finder?	○	○	○	○	○	○
Know how to access proteomic data, e.g., in NCBI protein databases?	○	○	○	○	○	○
Be able to use bioinformatics tools to examine protein structure and function, e.g., BLASTP, Cn3D, PyMol?	○	○	○	○	○	○
Know how to access metabolomic and systems biology data, e.g., in the Human Metabolome Database?	○	○	○	○	○	○

	Not at all important	Slightly important	Moderately important	Very important	Extremely important	× No opinion
Be able to use bioinformatics tools to examine the flow of molecules within pathways/networks, e.g., Gene Ontology, KEGG?	○	○	○	○	○	○
Be able to use bioinformatics tools to examine metagenomics data, e.g., MEGA, MUSCLE?	○	○	○	○	○	○

	Not at all important	Slightly important	Moderately important	Very important	Extremely important	× No opinion
Know how to write short computer programs as part of the scientific discovery process, e.g., write a script to analyze sequence data?	○	○	○	○	○	○
Be able to use software packages to manipulate and analyze bioinformatics data, e.g., Geneious, Vector NTI Express, spreadsheets?	○	○	○	○	○	○
Operate in a variety of computational environments to manipulate and analyze bioinformatics data, e.g., Mac OS, Windows, web- or cloud-based, Unix/Linux command line?	○	○	○	○	○	○

Q50

If there are bioinformatics competencies you feel are missing in the above, please describe them here.

Display This Question:

If Please select the statement below that best describes your current teaching of bioinformatics con... I teach dedicated bioinformatics course(s) to undergraduates majoring in life sciences or closely related disciplines. Is Selected

Or Please select the statement below that best describes your current teaching of bioinformatics con... I teach undergraduates majoring in life sciences or closely related disciplines and include substantial bioinformatics (more than one lecture/lab section) in my teaching. Is Selected

Or Please select the statement below that best describes your current teaching of bioinformatics con... I teach undergraduates majoring in life sciences or closely related disciplines and DO NOT currently include substantial bioinformatics (more than one lecture/lab section) in my teaching but will/would like to do so in the future. Is Selected

Q29

At your current institution, do you face any technical barriers in teaching bioinformatics, e.g., availability of a computer lab, different operating systems, access to high performance computing for teaching, IT support?

- Yes
- No

Display This Question:

If At your current institution, do you face any technical barriers in teaching bioinformatics, e.g.,... Yes Is Selected

Q30

Optional: Please describe.

Q52

In your opinion, how important is it for undergraduates majoring in life sciences or closely related disciplines to...

	Not at all important	Slightly important	Moderately important	Very important	Extremely important	× No opinion
Be familiar with the elements of computer programming, e.g., by way of a semester course in computer science?	○	○	○	○	○	○
Have a working knowledge of statistics, e.g., by way of a semester course in statistics or biostatistics?	○	○	○	○	○	○

Page Break

Q19

ABOUT YOU

Q14

Sex

- Female
- Male
- Rather not say

Q15

Race

- American Indian or Alaska Native
- Asian
- Black or African American
- Native Hawaiian or Other Pacific Islander
- White
- Rather not say

Q16

Ethnicity

- Hispanic or Latino
- Not Hispanic or Latino
- Rather not say

Q17

Highest earned degree. If "other," please explain.

- B.S. (or equivalent)
- M.S. (or equivalent)
- Professional degree (e.g., M.D.)
- Ph.D. (or equivalent)
- Other, please explain:

Q18

Year of highest earned degree.

2016

Q3

Which of the following best describes your level of bioinformatics training?

- No training/experience
- No formal training (self-taught)
- Short workshop/bootcamp
- Some undergraduate courses
- Undergraduate certificate
- Undergraduate degree
- Post-graduate certificate
- Graduate courses
- Graduate degree

Page Break

Display This Question:

If Please select the statement below that best describes your current teaching of bioinformatics con... I teach dedicated bioinformatics course(s) to undergraduates majoring in life sciences or closely related disciplines. Is Selected

Or Please select the statement below that best describes your current teaching of bioinformatics con... I teach undergraduates majoring in life sciences or closely related disciplines and include substantial bioinformatics (more than one lecture/lab section) in my teaching. Is Selected

Or Please select the statement below that best describes your current teaching of bioinformatics con... I teach undergraduates majoring in life sciences or closely related disciplines and DO NOT currently include substantial bioinformatics (more than one lecture/lab section) in my teaching but will/would like to do so in the future. Is Selected

Or Please select the statement below that best describes your current teaching of bioinformatics con... I supervise graduate students in the life sciences or closely related disciplines and expect, or would like to expect, graduate student familiarity with bioinformatics. Is Selected

Or To begin the survey, please select the statement that best describes your view of bioinformatics... I do not think bioinformatics should be integrated into undergraduate life sciences education. Is Selected

Q20

ABOUT YOUR INSTITUTION

Display This Question:

If Please select the statement below that best describes your current teaching of bioinformatics con... I teach dedicated bioinformatics course(s) to undergraduates majoring in life sciences or closely related disciplines. Is Selected

Or Please select the statement below that best describes your current teaching of bioinformatics con... I teach undergraduates majoring in life sciences or closely related disciplines and include substantial bioinformatics (more than one lecture/lab section) in my teaching. Is Selected

Or Please select the statement below that best describes your current teaching of bioinformatics con... I teach undergraduates majoring in life sciences or closely related disciplines and DO NOT currently include substantial bioinformatics (more than one lecture/lab section) in my teaching but will/would like to do so in the future. Is Selected

Or Please select the statement below that best describes your current teaching of bioinformatics con... I supervise graduate students in the life sciences or closely related disciplines and expect, or would like to expect, graduate student familiarity with bioinformatics. Is Selected

Or To begin the survey, please select the statement that best describes your view of bioinformatics... I do not think bioinformatics should be integrated into undergraduate life sciences education. Is Selected

Q21

What is the Carnegie classification of your institution?

- Associate's College
- Baccalaureate College
- Master's (Small, Medium, Large)
- Doctoral University (High, Higher, Highest Research Activity)
- Don't know

Display This Question:

If Please select the statement below that best describes your current teaching of bioinformatics con... I teach dedicated bioinformatics course(s) to undergraduates majoring in life sciences or closely related disciplines. Is Selected

Or Please select the statement below that best describes your current teaching of bioinformatics con... I teach undergraduates majoring in life sciences or closely related disciplines and include substantial bioinformatics (more than one lecture/lab section) in my teaching. Is Selected

Or Please select the statement below that best describes your current teaching of bioinformatics con... I teach undergraduates majoring in life sciences or closely related disciplines and DO NOT currently include substantial bioinformatics (more than one lecture/lab section) in my teaching but will/would like to do so in the future. Is Selected

Or Please select the statement below that best describes your current teaching of bioinformatics con... I supervise graduate students in the life sciences or closely related disciplines and expect, or would like to expect, graduate student familiarity with bioinformatics. Is Selected

Or To begin the survey, please select the statement that best describes your view of bioinformatics... I do not think bioinformatics should be integrated into undergraduate life sciences education. Is Selected

Q22

Is your institution classified as minority-serving?

- Yes
- No
- Don't know

Display This Question:

If Please select the statement below that best describes your current teaching of bioinformatics con... I teach dedicated bioinformatics course(s) to undergraduates majoring in life sciences or closely related disciplines. Is Selected

Or Please select the statement below that best describes your current teaching of bioinformatics con... I teach undergraduates majoring in life sciences or closely related disciplines and include substantial bioinformatics (more than one lecture/lab section) in my teaching. Is Selected

Or Please select the statement below that best describes your current teaching of bioinformatics con... I teach undergraduates majoring in life sciences or closely related disciplines and DO NOT currently include substantial bioinformatics (more than one lecture/lab section) in my teaching but will/would like to do so in the future. Is Selected

Or Please select the statement below that best describes your current teaching of bioinformatics con... I supervise graduate students in the life sciences or closely related disciplines and expect, or would like to expect, graduate student familiarity with bioinformatics. Is Selected

Or To begin the survey, please select the statement that best describes your view of bioinformatics... I do not think bioinformatics should be integrated into undergraduate life sciences education. Is Selected

Q23

What is the total number of students (undergraduate and graduate) at your institution?

- < 5,000 students
- 5,000 - 15,000 students
- > 15,000 students
- Don't know

Display This Question:

If Please select the statement below that best describes your current teaching of bioinformatics con... I teach dedicated bioinformatics course(s) to undergraduates majoring in life sciences or closely related disciplines. Is Selected

Or Please select the statement below that best describes your current teaching of bioinformatics con... I teach undergraduates majoring in life sciences or closely related disciplines and include substantial bioinformatics (more than one lecture/lab section) in my teaching. Is Selected

Or Please select the statement below that best describes your current teaching of bioinformatics con... I teach undergraduates majoring in life sciences or closely related disciplines and DO NOT currently include substantial bioinformatics (more than one lecture/lab section) in my teaching but will/would like to do so in the future. Is Selected

Or Please select the statement below that best describes your current teaching of bioinformatics con... I supervise graduate students in the life sciences or closely related disciplines and expect, or would like to expect, graduate student familiarity with bioinformatics. Is Selected

Or To begin the survey, please select the statement that best describes your view of bioinformatics... I do not think bioinformatics should be integrated into undergraduate life sciences education. Is Selected

Q24

What is the total number of undergraduate students at your institution?

- < 5,000 students
- 5,000 - 15,000 students
- > 15,000 students
- Don't know

Display This Question:

If Please select the statement below that best describes your current teaching of bioinformatics con... I teach dedicated bioinformatics course(s) to undergraduates majoring in life sciences or closely related disciplines. Is Selected

Or Please select the statement below that best describes your current teaching of bioinformatics con... I teach undergraduates majoring in life sciences or closely related disciplines and include substantial bioinformatics (more than one lecture/lab section) in my teaching. Is Selected

Or Please select the statement below that best describes your current teaching of bioinformatics con... I teach undergraduates majoring in life sciences or closely related disciplines and DO NOT currently include substantial bioinformatics (more than one lecture/lab section) in my teaching but will/would like to do so in the future. Is Selected

Or Please select the statement below that best describes your current teaching of bioinformatics con... I supervise graduate students in the life sciences or closely related disciplines and expect, or would like to expect, graduate student familiarity with bioinformatics. Is Selected

Or To begin the survey, please select the statement that best describes your view of bioinformatics... I do not think bioinformatics should be integrated into undergraduate life sciences education. Is Selected

Q25

What is the name of your department/unit (e.g., Department of Biology, Biochemistry Department, School of Interdisciplinary Informatics)?

Display This Question:

If Please select the statement below that best describes your current teaching of bioinformatics con... I teach dedicated bioinformatics course(s) to undergraduates majoring in life sciences or closely related disciplines. Is Selected

Or Please select the statement below that best describes your current teaching of bioinformatics con... I teach undergraduates majoring in life sciences or closely related disciplines and include substantial bioinformatics (more than one lecture/lab section) in my teaching. Is Selected

Or Please select the statement below that best describes your current teaching of bioinformatics con... I teach undergraduates majoring in life sciences or closely related disciplines and DO NOT currently include substantial bioinformatics (more than one lecture/lab section) in my teaching but will/would like to do so in the future. Is Selected

Or Please select the statement below that best describes your current teaching of bioinformatics con... I supervise graduate students in the life sciences or closely related disciplines and expect, or would like to expect, graduate student familiarity with bioinformatics. Is Selected

Or To begin the survey, please select the statement that best describes your view of bioinformatics... I do not think bioinformatics should be integrated into undergraduate life sciences education. Is Selected

Q26

How many full-time faculty are in your department/unit? (Do not include part-time faculty or adjuncts.)

- < 10
- 10 - 20
- 21 - 30
- 31 - 40
- 41 - 50
- > 50
- Don't know

Display This Question:

If Please select the statement below that best describes your current teaching of bioinformatics con... I teach dedicated bioinformatics course(s) to undergraduates majoring in life sciences or closely related disciplines. Is Selected

Or Please select the statement below that best describes your current teaching of bioinformatics con... I teach undergraduates majoring in life sciences or closely related disciplines and include substantial bioinformatics (more than one lecture/lab section) in my teaching. Is Selected

Or Please select the statement below that best describes your current teaching of bioinformatics con... I teach undergraduates majoring in life sciences or closely related disciplines and DO NOT currently include substantial bioinformatics (more than one lecture/lab section) in my teaching but will/would like to do so in the future. Is Selected

Or Please select the statement below that best describes your current teaching of bioinformatics con... I supervise graduate students in the life sciences or closely related disciplines and expect, or would like to expect, graduate student familiarity with bioinformatics. Is Selected

Or To begin the survey, please select the statement that best describes your view of bioinformatics... I do not think bioinformatics should be integrated into undergraduate life sciences education. Is Selected

Q27

How many undergraduate students are in your department/unit (all majors)?

- < 50
- 51 - 100
- 101 - 500
- 501 - 2000
- > 2000
- Don't know

Page Break

Display This Question:

If Please select the statement below that best describes your current teaching of bioinformatics con... I teach dedicated bioinformatics course(s) to undergraduates majoring in life sciences or closely related disciplines. Is Selected

Or Please select the statement below that best describes your current teaching of bioinformatics con... I teach undergraduates majoring in life sciences or closely related disciplines and include substantial bioinformatics (more than one lecture/lab section) in my teaching. Is Selected

Q28

For each undergraduate course you teach that includes bioinformatics content, please provide the name of the course, the department/unit(s) in which the course is listed, and a brief description of the course.

Display This Question:

If Please select the statement below that best describes your current teaching of bioinformatics con... I teach dedicated bioinformatics course(s) to undergraduates majoring in life sciences or closely related disciplines. Is Selected

Or Please select the statement below that best describes your current teaching of bioinformatics con... I teach undergraduates majoring in life sciences or closely related disciplines and include substantial bioinformatics (more than one lecture/lab section) in my teaching. Is Selected

Or Please select the statement below that best describes your current teaching of bioinformatics con... I teach undergraduates majoring in life sciences or closely related disciplines and DO NOT currently include substantial bioinformatics (more than one lecture/lab section) in my teaching but will/would like to do so in the future. Is Selected

Or Please select the statement below that best describes your current teaching of bioinformatics con... I supervise graduate students in the life sciences or closely related disciplines and expect, or would like to expect, graduate student familiarity with bioinformatics. Is Selected

Q33

In your opinion, what do you think are the most important challenges currently facing those educating undergraduate life scientists in bioinformatics?

Display This Question:

If Please select the statement below that best describes your current teaching of bioinformatics con... I teach dedicated bioinformatics course(s) to undergraduates majoring in life sciences or closely related disciplines. Is Selected

And As part of our work, we are building an online repository of bioinformatics syllabi and content a... No Is Not Selected

Or Please select the statement below that best describes your current teaching of bioinformatics con... I teach undergraduates majoring in life sciences or closely related disciplines and include substantial bioinformatics (more than one lecture/lab section) in my teaching. Is Selected

And As part of our work, we are building an online repository of bioinformatics syllabi and content a... No Is Not Selected

Q39

As part of our work, we are building an online repository of bioinformatics content assessments and syllabi of dedicated bioinformatics courses and life sciences courses with bioinformatics content. Earlier in the survey you indicated a willingness to share your syllabi and/or assessments for this purpose. Please provide those here.

To preserve the confidentiality of your survey response, the survey administrator will disassociate any linked or uploaded files from your survey response before sharing these with the research team for the purpose of the repository.

ENTER URL LINKS TO ANY CONTENT YOU ARE WILLING TO SHARE HERE:

Display This Question:

If Please select the statement below that best describes your current teaching of bioinformatics con... I teach dedicated bioinformatics course(s) to undergraduates majoring in life sciences or closely related disciplines. Is Selected

And As part of our work, we are building an online repository of bioinformatics syllabi and content a... No Is Not Selected

Or Please select the statement below that best describes your current teaching of bioinformatics con... I teach undergraduates majoring in life sciences or closely related disciplines and include substantial bioinformatics (more than one lecture/lab section) in my teaching. Is Selected

And As part of our work, we are building an online repository of bioinformatics syllabi and content a... No Is Not Selected

Q44

UPLOAD COURSE SYLLABI
Please securely upload your relevant course syllabi below. Please note that the system is only able to accept one file upload at a time (max size 16 MB), so if you have multiple syllabi to upload, please combine them prior to upload.

Choose File No file chosen

Display This Question:

If Please select the statement below that best describes your current teaching of bioinformatics con... I teach dedicated bioinformatics course(s) to undergraduates majoring in life sciences or closely related disciplines. Is Selected

And As part of our work, we are building an online repository of bioinformatics syllabi and content a... No Is Not Selected

Or Please select the statement below that best describes your current teaching of bioinformatics con... I teach undergraduates majoring in life sciences or closely related disciplines and include substantial bioinformatics (more than one lecture/lab section) in my teaching. Is Selected

And As part of our work, we are building an online repository of bioinformatics syllabi and content a... No Is Not Selected

Q34

UPLOAD CONTENT ASSESSMENTS
Please securely upload your relevant content assessments (e.g., quiz and exam questions) below. Please note that the system is only able to accept one file upload (max size 16 MB) at a time, so if you have multiple assessments to upload, please combine them prior to upload.

Choose File No file chosen

Display This Question:

If Please select the statement below that best describes your current teaching of bioinformatics con... I supervise graduate students in the life sciences or closely related disciplines and expect, or would like to expect, graduate student familiarity with bioinformatics. Is Selected

Q41

In your opinion, what bioinformatics skill(s) are incoming graduate students most deficient in?

Q35

Additional comments:

Add Block

End of Survey

Survey Termination Options...
