# Supplementary material for: Bioinformatics core competencies for undergraduate life sciences education
Source: PLoS One. 2018 Jun 5;13(6):e0196878. doi: 10.1371/journal.pone.0196878 (PMC5988330; doi:10.1371/journal.pone.0196878)
Supplement: S2 Survey — In this view, only the survey questions are given—the branching structure of the survey is not evident. (PDF) [file pone.0196878.s002.pdf]

Default Question Block

Dear Life Sciences Educator,

The Network for Integrating Bioinformatics into Life Sciences Education (NIBLSE; "nibbles") is a National Science Foundation Research Coordination Network for Undergraduate Biology Education (RCN-UBE) devoted to establishing bioinformatics as essential to the undergraduate life sciences curriculum. To that end, we are asking the community to help us determine core bioinformatics competencies for the undergraduate curriculum.

We are asking you to complete a short, anonymous survey if you are in one or more of the following groups:

- Educators who teach undergraduate life sciences at a 2-year or 4-year college, university, or technical school.
- Educators who supervise graduate students and who expect, or would like to expect, graduate student familiarity with bioinformatics.
- Biologists and/or bioinformaticians who teach/provide training in bioinformatics as part of their work at a company or organization, but not as part of a for-credit course at a college or university.

The survey should take you approximately 15 minutes to complete.

We invite you to read more about our activities and other ways to contribute and provide feedback at our project website or contact us at the address below. Thank you in advance for your input.

NIBLSE Leadership Team:
Mark Pauley (mark@niblse.org), University of Nebraska at Omaha
Elizabeth Dinsdale, San Diego State University
William Morgan, College of Wooster
Anne Rosenwald, Georgetown University
Eric Triplett, University of Florida

This survey is covered by IRB 161-16-EX. The survey administrator will disassociate any linked or uploaded files from your survey response before sharing these with the research team. For questions about the survey, please contact Mindy McWilliams. NIBLSE is supported by NSF Award #1539900.

NIBLSE is a proud partner of QUBES: https://qubeshub.org/

I agree to participate.

I do not agree to participate.

To begin the survey, please select the statement that best describes your view of bioinformatics in undergraduate life sciences education.

I think bioinformatics should be integrated into undergraduate life sciences education.

I do not think bioinformatics should be integrated into undergraduate life sciences education.

Please select the statement below that best describes you.

I teach at a 4-year college or university.

I teach at a 2-year college or technical school.

I teach/provide training in bioinformatics as a regular part of my work at a company or organization, but not as part of a for-credit course at a college or university.

Please select the statement below that best describes your current teaching of bioinformatics content at your institution.

I teach dedicated bioinformatics course(s) to undergraduates majoring in life sciences or closely related disciplines.

I teach undergraduates majoring in life sciences or closely related disciplines and include substantial bioinformatics (more than one lecture/lab section) in my teaching.

I teach undergraduates majoring in life sciences or closely related disciplines and DO NOT currently include substantial bioinformatics (more than one lecture/lab section) in my teaching but will/would like to do so in the future.

I supervise graduate students in the life sciences or closely related disciplines and expect, or would like to expect, graduate student familiarity with bioinformatics.

ABOUT YOUR CURRICULUM

What is the level of the dedicated bioinformatics course(s) you teach? (Check all that apply.)

Freshman

Sophomore

Junior

Senior

What is the level of the courses with bioinformatics content that you teach? (Check all that apply.)

Freshman

Sophomore

Junior

Senior

What is the level of the course(s) you teach? (Check all that apply.)

Freshman

Sophomore

What is the level of the courses you teach in which you would like to include bioinformatics content? (Check all that apply.)

Freshman

Sophomore

As part of our work, we are building an online repository of bioinformatics syllabi and content assessments. Would you be willing to share your syllabus/syllabi and/or content assessment(s) with us so they could be added to this repository?

Yes

Maybe

No

What is the level of the courses you teach in which you would like to include bioinformatics content? (Check all that apply.)

Freshman

Sophomore

Junior

Senior

What is preventing you from including bioinformatics content in these courses?

Are there undergraduate courses with bioinformatics content at your institution that life sciences students routinely take? If "yes," please encourage those teaching these classes to complete the survey (forward the solicitation e-mail).

Yes

No

Don't know

Aside from the course(s) you teach, are there other undergraduate courses with bioinformatics content at your institution that life sciences students routinely take? If “yes,” please encourage those teaching these courses to complete this survey (forward the solicitation e-mail).

Yes

No

Don't know

In your opinion, are additional undergraduate courses with bioinformatics content needed at your institution?

Yes

No

Don't know

Optional: Please describe briefly; include any barriers to development and/or implementation.

Is an undergraduate bioinformatics certificate or minor offered at your institution?

Yes

No

Don't know

Optional: Please give certificate or minor name, department/unit in which it's

offered, and website URL (if available).

Is an undergraduate bioinformatics major offered at your institution?

Yes

No

Don't know

Optional: Please give name, department/unit in which it's offered, and website URL (if available):

ABOUT YOUR TRAINING

Briefly describe the format of the bioinformatics training (e.g., boot camp, short course, etc.) you most commonly provide.

Briefly describe your audience for this training.

In your opinion, what are the biggest bioinformatics needs of those taking your training?

What reasons do your students provide as to why they are taking your training (e.g., professional advancement, have a research problem they need to solve, have been tasked with teaching a course in bioinformatics)?

BIOINFORMATICS COMPETENCIES FOR UNDERGRADUATE LIFE SCIENTISTS

In your opinion, how important is it for undergraduates majoring in life sciences or closely related disciplines to...

	Not at all important	Slightly important	Moderately important	Very important	Extremely important	No opinion
Understand the role of computation and data mining in hypothesis-driven processes within the life sciences?	O	O	O	O	O	O
Understand computational concepts used in bioinformatics, e.g., meaning of algorithm, bioinformatics file formats?	O	O	O	O	O	O
Know statistical						

	Not at all important	Slightly important	Moderately important	Very important	Extremely important	No opinion
concepts used in bioinformatics, e.g., E-value, z-scores, t-test?	○	○	○	○	○	○
Know how to access genomic data, e.g., in NCBI nucleotide databases?	○	○	○	○	○	○
Be able to use bioinformatics tools to analyze genomic data, e.g., BLASTN, genome browser?	○	○	○	○	○	○

	Not at all important	Slightly important	Moderately important	Very important	Extremely important	No opinion
Know how to access gene expression data, e.g., in UniGene, GEO, SRA?	○	○	○	○	○	○
Be able to use bioinformatics tools to analyze gene expression data, e.g., GeneSifter, David, ORF Finder?	○	○	○	○	○	○
Know how to access proteomic data, e.g., in NCBI protein databases?	○	○	○	○	○	○
Be able to use bioinformatics tools to examine protein structure and function, e.g., BLASTP, Cn3D, PyMol?	○	○	○	○	○	○
Know how to access metabolomic and systems biology data, e.g., in the Human Metabolome Database?	○	○	○	○	○	○

	Not at all important	Slightly important	Moderately important	Very important	Extremely important	No opinion
Be able to use bioinformatics tools to examine the flow of molecules within pathways/networks, e.g., Gene Ontology, KEGG?	O	O	O	O	O	O
Be able to use bioinformatics tools to examine metagenomics data, e.g., MEGA, MUSCLE?	O	O	O	O	O	O
Know how to write short computer programs as part of the scientific discovery process, e.g., write a script to analyze sequence data?	O	O	O	O	O	O
Be able to use software packages to manipulate and analyze bioinformatics data, e.g., Geneious, Vector NTI Express, spreadsheets?	O	O	O	O	O	O
Operate in a variety of computational environments to manipulate and analyze bioinformatics data, e.g., Mac OS, Windows, web- or cloud-based, Unix/Linux command line?	O	O	O	O	O	O

If there are bioinformatics competencies you feel are missing in the above, please describe them here.

At your current institution, do you face any technical barriers in teaching bioinformatics, e.g., availability of a computer lab, different operating systems, access to high performance computing for teaching, IT support?

Yes

No

Optional: Please describe.

In your opinion, how important is it for undergraduates majoring in life sciences or closely related disciplines to...

	Not at all important	Slightly important	Moderately important	Very important	Extremely important	No opinion
Be familiar with the elements of computer programming, e.g., by way of a semester course in computer science?	○	○	○	○	○	○
Have a working knowledge of statistics, e.g., by way of a semester course in statistics or biostatistics?	○	○	○	○	○	○

ABOUT YOU

Sex

Female

Male

Rather not say

Race

American Indian or Alaska Native

Asian

Black or African American

Native Hawaiian or Other Pacific Islander

White

Rather not say

Ethnicity

Hispanic or Latino

Not Hispanic or Latino

Rather not say

Highest earned degree. If "other," please explain.

B.S. (or equivalent)

M.S. (or equivalent)

Professional degree (e.g., M.D.)

Ph.D. (or equivalent)

Other, please explain:

Year of highest earned degree.

Which of the following best describes your level of bioinformatics training?

No training/experience
No formal training (self-taught)
Short workshop/bootcamp
Some undergraduate courses
Undergraduate certificate
Undergraduate degree
Post-graduate certificate
Graduate courses
Graduate degree

ABOUT YOUR INSTITUTION

What is the Carnegie classification of your institution?

Associate's College
Baccalaureate College
Master's (Small, Medium, Large)
Doctoral University (High, Higher, Highest Research Activity)
Don't know

Is your institution classified as minority-serving?

Yes
No
Don't know

What is the total number of students (undergraduate and graduate) at your institution?

< 5,000 students
5,000 - 15,000 students
> 15,000 students
Don't know

What is the total number of undergraduate students at your institution?

< 5,000 students

5,000 - 15,000 students

> 15,000 students

Don't know

What is the name of your department/unit (e.g., Department of Biology, Biochemistry Department, School of Interdisciplinary Informatics)?

How many full-time faculty are in your department/unit? (Do not include part-time faculty or adjuncts.)

< 10

10 - 20

21 - 30

31 - 40

41 - 50

> 50

Don't know

How many undergraduate students are in your department/unit (all majors)?

< 50

51 - 100

101 - 500

501 - 2000

> 2000

Don't know

For each undergraduate course you teach that includes bioinformatics content, please provide the name of the course, the department/unit(s) in which the course is listed, and a brief description of the course.

In your opinion, what do you think are the most important challenges currently facing those educating undergraduate life scientists in bioinformatics?

As part of our work, we are building an online repository of bioinformatics content assessments and syllabi of dedicated bioinformatics courses and life sciences courses with bioinformatics content. Earlier in the survey you indicated a willingness to share your syllabi and/or assessments for this purpose. Please provide those here.

To preserve the confidentiality of your survey response, the survey administrator will disassociate any linked or uploaded files from your survey response before sharing these with the research team for the purpose of the repository.

ENTER URL LINKS TO ANY CONTENT YOU ARE WILLING TO SHARE HERE:

UPLOAD COURSE SYLLABI

Please securely upload your relevant course syllabi below. Please note that the system is only able to accept one file upload at a time (max size 16 MB), so if you have multiple syllabi to upload, please combine them prior to upload.

UPLOAD CONTENT ASSESSMENTS

Please securely upload your relevant content assessments (e.g., quiz and exam questions) below. Please note that the system is only able to accept one file upload (max size 16 MB) at a time, so if you have multiple assessments to upload, please combine them prior to upload.

In your opinion, what bioinformatics skill(s) are incoming graduate students most deficient in?

Additional comments:
