# Supplementary material for: Bioinformatics core competencies for undergraduate life sciences education
Source: PLoS One. 2018 Jun 5;13(6):e0196878. doi: 10.1371/journal.pone.0196878 (PMC5988330; doi:10.1371/journal.pone.0196878)
Supplement: S1 Table — Means of the Likert-scale responses for respondents whose home institution is classified as Associate’s (Assc), Baccalaureate (BS), Master’s (MS), or Doctoral (PhD). Two-sided P values from a Kolmogorov-Smirnov test of the Likert-scale responses for each pairwise test are shown. The pairs are indicated by the heading of the column; e.g., P_Assc_BS is the P value for the Associate’s (Assc)/Baccalaureate (BS) pair. Significant differences are in bold. (DOCX) [file pone.0196878.s003.docx]

| **Skill** | **Assc** | **BS** | **MS** | **PhD** | ***P*_Assc_BS** | ***P*_Assc_MS** | ***P*_Assc_PhD** | ***P*_BS_MS** | ***P*_BS_PhD** | ***P*_MS_PhD** |
| --- | --- | --- | --- | --- | --- | --- | --- | --- | --- | --- |
| S1 | 3.937 | 4.071 | 4.144 | 4.285 | 0.7634 | 0.2062 | **0.0215** | 0.9847 | **0.0227** | 0.1252 |
| S2 | 3.127 | 3.24 | 3.459 | 3.618 | 0.9725 | 0.0739 | **0.0002** | 0.1919 | **0.0006** | 0.3329 |
| S3 | 3.66 | 4.004 | 4.073 | 4.118 | 0.2647 | **0.0237** | **0.0006** | 0.8908 | 0.2082 | 0.9984 |
| S4 | 3.937 | 4.348 | 4.365 | 4.222 | **0.0059** | **0.0104** | 0.0692 | 0.9999 | 0.8539 | 0.6417 |
| S5 | 3.787 | 4.207 | 4.219 | 4.1 | **0.0193** | **0.0090** | 0.0588 | 1.0000 | 0.9919 | 0.9335 |
| S6 | 3.284 | 3.402 | 3.455 | 3.415 | 0.9240 | 0.7256 | 0.9893 | 0.8989 | 0.7463 | 0.9971 |
| S7 | 3.115 | 3.381 | 3.398 | 3.283 | 0.1260 | 0.1725 | 0.4016 | 0.9490 | 0.4904 | 0.9816 |
| S8 | 3.461 | 3.716 | 3.778 | 3.609 | 0.3526 | 0.1760 | 0.8843 | 0.1671 | 0.3987 | 0.7214 |
| S9 | 3.344 | 3.611 | 3.606 | 3.439 | 0.0881 | 0.3552 | 0.9302 | 1.0000 | 0.0686 | 0.3931 |
| S10 | 3.117 | 3.145 | 3.19 | 3.116 | 0.6546 | 0.7620 | 1.0000 | 1.0000 | 0.3707 | 0.5031 |
| S11 | 3 | 3.148 | 3.271 | 3.248 | 0.3277 | 0.1056 | 0.3168 | 0.9654 | 0.4765 | 0.9938 |
| S12 | 2.881 | 3.095 | 3.235 | 3.189 | **0.0105** | **0.0441** | **0.0493** | 0.3499 | 0.6142 | 0.9997 |
| S13 | 2.267 | 2.793 | 2.794 | 3.18 | **0.0001** | **0.0004** | **1.2926 × 10^-10^** | 1.0000 | **0.0008** | **0.0134** |
| S14 | 2.926 | 3.183 | 3.37 | 3.401 | **0.0285** | **0.0064** | **0.0050** | 0.3410 | **0.0234** | 0.9969 |
| S15 | 2.856 | 3.099 | 3.206 | 3.44 | 0.1024 | 0.0669 | **1.8909 × 10^-5^** | 0.9425 | **0.0171** | 0.2261 |
